# A Case of Paralysis of the Bladder Terminating Fatally

**Published:** 1840-07

**Authors:** F. Walton Todd

**Affiliations:** Port-Gibson, Mississippi


**Art. IV.**—*A Case of Paralysis of the Bladder terminating fatally.* By F. Walton Todd, M.D., of Port-Gibson, Mississippi.

Dr. W. was called a few days since to see a negro man, who being delirious was unable to say what was the matter with him; and though he had a family it was not ascertained, that for several days he had not evacuated his urine. He seemed to be suffering intensely, and, in a way which was not intelligible, referred to some abdominal distress. Upon examination it was thought, that there was a preternatural distention of the urinary visicle, and a flexible catheter was introduced without difficulty; but no urine passed. A metalic instrument was then used, but with the same result; he was finally placed so that the water would gravitate, but all without effect. In a few hours he died, and upon examination the bladder was found immensely distended, with a thin, not unnatural fluid; the catheter which had been introduced was found to have penetrated the bladder successfully, which contained four or five pints of water. There were no traces of inflammation; no morbid appearances, within the bladder, prostrate gland, or urethra; no obstruction in the urinary passages, but a perfectly normal condition of the internal coat of the bladder and urethra. I look upon this case as one confirming the fact, that in paralysis of the bladder urine will often not pass by the catheter, without grasping and compressing the visicle, and sometimes cannot be drawn off except by paracentisis. I have since seen an analogous case reported by a Parisian writer to the Boston Medical and Surgical Journal, where M. Cruveilhier had no better success with the catheter unaided by compression.

*June*, 1840.

